# Retraction: Clinical impact of monocyte distribution width and neutrophil-to-lymphocyte ratio for distinguishing COVID-19 and influenza from other upper respiratory tract infections: A pilot study

**DOI:** 10.1371/journal.pone.0326523

**Published:** 2025-06-18

**Authors:** The PLOS One Editors

This article [1] was republished on January 14, 2025, to address issues identified post-publication. In addition, during post-publication assessment, PLOS identified discrepancies between the work reported in [1] and the study protocol and consent procedures approved by the Joint Institutional Review Board of Taipei Medical University (TMU-JIRB) under approval number: N201904066 reported in [1]. In editorial follow-up, the corresponding author stated that COVID-19 patients were enrolled within the IRB-approved framework as a subset of patients meeting the original inclusion criteria and that amended restrictions during the COVID-19 outbreak allowed publication of research findings based on data already collected under emergency authorization. The Joint Institutional Review Board of Taipei Medical University (TMU-JIRB) stated that the study reported in [1] did not have appropriate ethical oversight, as the ethics approval documents, protocol, and consent form do not cover the work in [1] relating to COVID-19 patients; IRB review and approval of protocol amendments or a new protocol were required but not obtained prior to study commencement.

In light of the concerns that this study was not conducted in line with institutional ethics policies and *PLOS One*’s Human Subject Research and Ethical Publishing Practice policies in place at the time of submission, the *PLOS One* Editors retract this article. PLOS regrets that these issues were not identified prior to the publication of the article.

SKH did not agree with the retraction and stands by the article’s findings. SFL responded but expressed neither agreement nor disagreement with the editorial decision. HAL, HWC, YJL, and RJC either did not respond directly or could not be reached.
